# Damage Characterization of Carbon Fiber Composite Pressure Vessels Based on Modal Acoustic Emission

**DOI:** 10.3390/ma15144783

**Published:** 2022-07-08

**Authors:** Peng Jiang, Xiaodong Liu, Wei Li, Fuping Guo, Chuan Hong, Yubin Liu, Chang Yang

**Affiliations:** 1 College of Mechanical Science and Engineering, Northeast Petroleum University, Daqing 163318, China; liuxiaodong961100@163.com (X.L.); liweinepu2021@163.com (W.L.); gfpmmc@163.com (F.G.); 17756522702@163.com (C.H.); lyb161101@163.com (Y.L.); yc081204@163.com (C.Y.); 2 College of Mechanical and Electrical Engineering, Guangdong University of Petrochemical Technology, Maoming 525000, China

**Keywords:** carbon fiber composite pressure vessel, MAE parameters, modal damage identification, damage evolution mechanisms

## Abstract

This study characterized the damage characteristics of carbon fiber composite pressure vessels by the modal acoustic emission (MAE) method. The study showed how to use the extracted damage modal features and established MAE parameters to determine the damage mode of composite pressure vessels. First, the A_0_ and S_0_ Lamb modes of the AE signal were split through mode separation, and the time window was selected to establish the MAE characteristic parameters. Subsequently, based on the MAE parameters and the damage mode characteristics established from single damage experiments, a damage mode discrimination method was established. A bending test of carbon fiber composite laminates proved that the modal separation method and the MAE parameters establishment are reasonable and effective. The results from the hydraulic test of the graded loading performed on 20 MPa carbon fiber composite pressure vessels showed the accuracy of the damage mode discrimination method, and the damage state of the pressure vessel could be analyzed using the fiber fracture damage threshold according to the MAE parameters.

## 1. Introduction

Composite structures are widely used in containers for transportation and energy storage and in oil and gas pipelines as well as other commercial areas. In automobile industries that use hydrogen energy, aluminum liner and plastic liner carbon fiber completely wound composite pressure vessels are commonly used for high-pressure hydrogen storage, due to their excellent material properties, such as outstanding strength, stiffness-to-weight ratio, fatigue performance, and corrosion resistance [1,2,3]. In addition, the design and manufacture of these vessels has adopted new and advanced filament winding technology, which further improves their material properties [4].

Numerous experiments and numerical simulations have shown that vessel failure is not abrupt [5,6,7,8]. Failure involves various failure types, such as matrix cracking, interface delamination, and fiber fracture, which lead to ultimate vessel failure. To ensure the safe utilization of composite pressure vessels, these vessels should be regularly analyzed and evaluated. New nondestructive examination (NDE) techniques have been successfully introduced as alternatives to the conventional retesting procedures of vessels, such as gas cylinders and tubes [9,10,11]. Acoustic emission (AE) testing, which has been proven to be an acceptable testing method during periodic inspections in several countries, is an alternative NDE technique for certain applications [12]. Researchers have conducted AE studies on composite pressure vessels [13,14,15]. The production quality of fiber-reinforced pressure vessels was monitored using AE testing on the production line [13]. The AE method was used to detect damage caused by constant and cyclic air pressure loads [14]. The thermal performance of fatigue cycling under hydraulic and air pressure was studied [15]. However, these studies have neither characterized the damage mode of composite vessels nor recognized the damage evolution mechanism.

Studies have shown that in composites, the characteristics of AE signals are associated with common damage mechanisms. This correlation can be selected or established to detect and locate the initiation and development of material damage, assess the overall damage severity of materials, and identify damage patterns [16,17,18,19,20]. Researchers have associated each injury mechanism with specific AE characteristic parameters (amplitude, count, and energy) [16,17,18]. Li et al. [19], based on the K-means algorithm, identified the damage mode of composite laminates. To improve the identification process of the damage mechanism, Marec [20] et al. used the fuzzy C-means clustering method combined with principal components to analyze the components, cluster the acoustic emission data, and correlate the clustering results with the damage mechanism. However, the use of these clustering methods also presents some disadvantages. In the K-means algorithm, the number of clusters to be clustered must be specified; this number is sensitive to the selection of initial values, and some outliers that are considerably large show a substantial impact on the results. Moreover, the characteristics of AE signals are easily affected by various wave propagation effects, such as reflection, attenuation, mode conversion, and dispersion.

Since the 1990s, in MAE, the theory of Lamb waves has been widely used to analyze AE signals, and MAE has become a promising technology [21,22,23]. According to theory of Lamb waves [24,25], AE waves propagate in two main modes in a plate: symmetric or extensional (S_0_) mode and anti-symmetric or flexural (A_0_) mode, and separating these modes can enable researchers to extract information about the damage mechanism. Thus, lamb waves with dispersion characteristics can provide a suitable method for the evaluation of the damage mechanism of composites [26,27,28,29]. For instance, Baker et al. [28] employed MAE and waveform energies coupled with peak frequency data and correlated to matrix crack density in the transverse direction; they studied the crack initiation and propagation of composite laminates. Dahmene et al. [23] used the MAE method to extract the modal of the AE signal during the tensile test. They found that the damage and dominant modes had a satisfactory correspondence but did not identify the main damage mode. José et al. [29] used the MAE method and achieved the real-time assessment of delaminated damage, by using an algorithm for comparing the intensities of the two modes, to associate the AE event with a specific damage mechanism. The damage mechanism of composite pressure vessels is consistent with that of composite laminates; thus, the damage modal analysis of composite vessels based on MAE is a promising method.

The main objective of this study was to use the MAE method to evaluate the damage of carbon fiber composite pressure vessels. The two modes (S_0_ and A_0_) and their frequency ranges were related to three damage mechanisms of composite materials (matrix cracking, delamination, and fiber fracture). On the basis of this phenomenon, a modal damage identification method was proposed. To verify the effectiveness of this method, hydraulic pressure tests of carbon fiber composite pressure vessels were conducted, and the evolution rules of different damage mechanisms were analyzed during compression.

## 2. Materials and Methods

In this study, experiments were mainly classified into three parts, namely pencil lead-breakage (PLB) tests, bending tests of carbon fiber composite laminate, and carbon fiber composite pressure vessels tests. PLB tests were performed on the carbon fiber composite laminate, which were used to verify that there is a modal effect in the composite acoustic emission signal. By using a hierarchical load mechanical testing machine for failure, the bending test proved that MAE technology can be used to characterize the damage evolution law of carbon fiber composites. Finally, the MAE analysis method proposed in this paper was applied to the damage evolution characterization and evaluation process of carbon fiber composite pressure vessels and verified. The AE monitoring system, which included the AE software AE-Win and the acquisition module for recording AE signals, that was used in all the experiments was provided by Physical Acoustics Corporation (PAC). For the method used, first, EMD’s inherent modal decomposition was effectively used to separate the modes, which was verified using PLB tests. Second, the method of establishing new parameters was employed to evaluate and analyze the in-plane damage of laminates. Finally, based on the damage modal characteristics extracted from three single damage tests, a method was established to distinguish the damage modes of composite vessels.

### 2.1. Preliminary Pencil Lead Breakage (PLB) Experiments to Verify the Modal Separation Method

**Figure 1:**
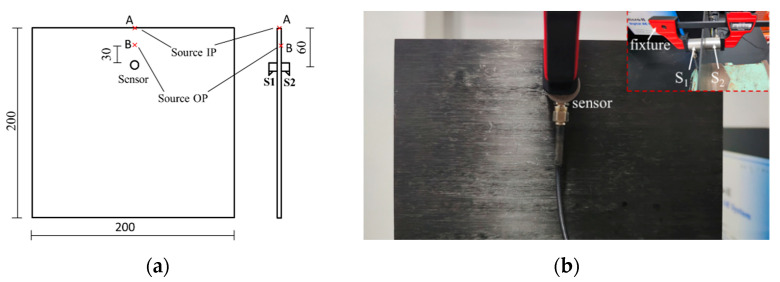
Mode detection experimental scheme (**a**) sensors layout diagram; (**b**) Distances in mm.

To verify the feasibility of the modal separation method, PLBs were performed on the 14-layer T700 carbon fiber composite laminate with a layer mode of [45°/−45°]_14_ and size of 200 mm× 200 mm× 2 mm (length × width × height) The AE sensor uses two WDI sensors with a frequency of 200–900 kHz. The surfaces of the sensor and laminate were coupled with vacuum grease, and each sensor was calibrated using pencil lead breakage tests to ensure successful signal acquisition. The pencil lead broke outside the plane (OP) and in the plane (IP), that are the positions of B and A in Figure 1a. Two sensors (S1 and S2) were located 60 mm from the top of the laminate, at the same point but on opposite sides of the plate, following the technique described in [30] (see Figure 1). The specific system configuration parameters are shown in Table 1.

### 2.2. In-Plane Bending Test of Carbon Fiber Composite Laminates

**Figure 2:**
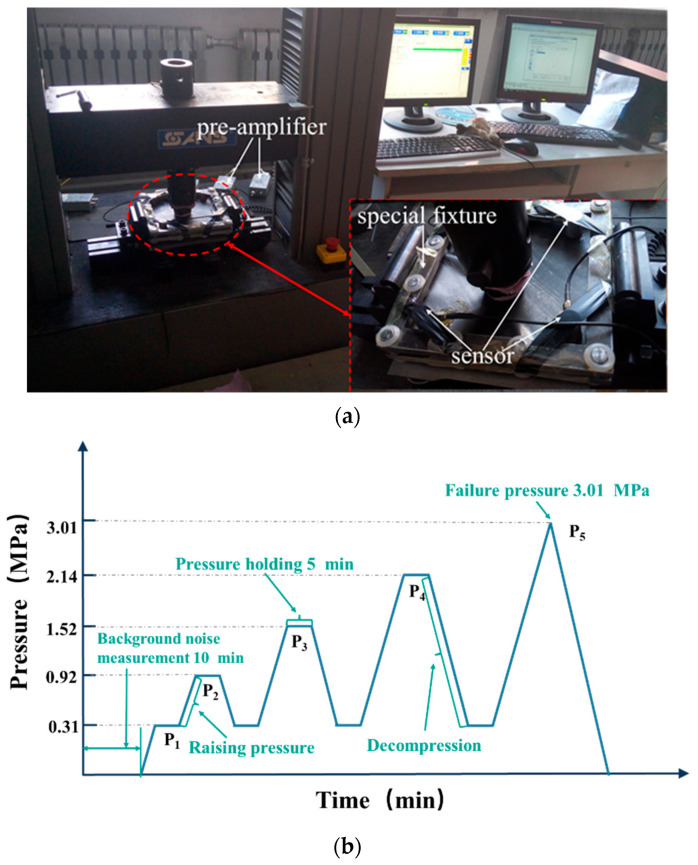
Bending damage AE emission monitoring (**a**), and loading curve of in-plane bending test (**b**).

The materials used in this experiment were the same as those used in the PLB experiment. The experiment used four WD sensors (the main frequency range was 125–1000 kHz). The rest of the AE equipment and signal acquisition settings were the same as those presented for the experiment in Section 2.1. The experimental device is shown in Figure 2a. Loading was performed with the SANS universal testing machine of Jinan MTS. The loading curve is shown in Figure 2b.

At room temperature, the speed of compressive loading of the sample was 1 mm/min. When the limit load was reached, the holding time of the constant pressure was 5 min, and it was then unloaded at a speed of 1 mm/min. According to the test results, the maximum failure load was 3.01 MPa, and the holding pressures of staged loading were P_1_–P_5_.

### 2.3. Damage Experiment of Carbon Fiber Composite Material Vessel

**Figure 3:**
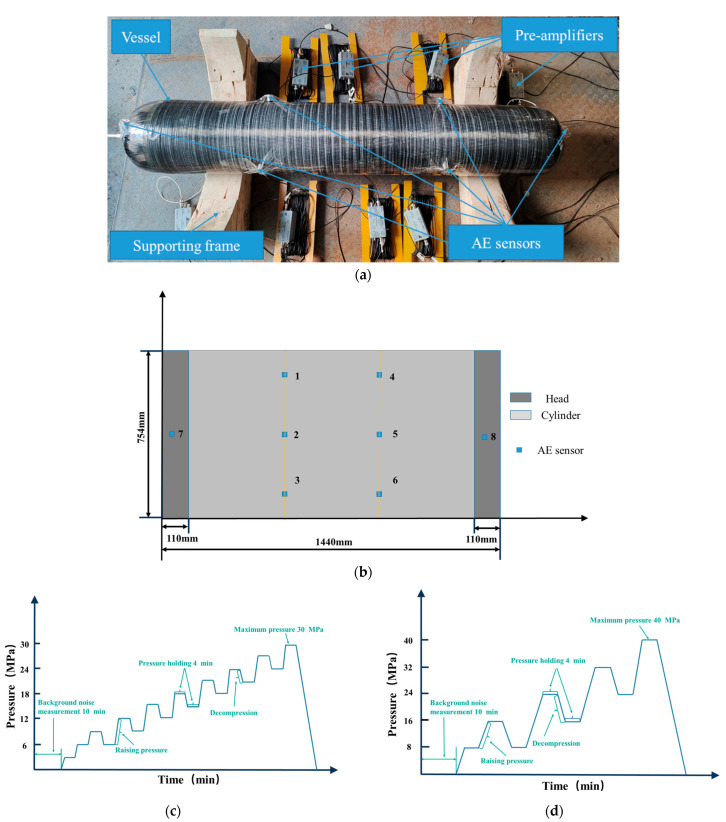
System connection of experimental device for the pressure vessel pressurization test (**a**), sensor layout diagram on the vessel (**b**), hydraulic time loading curve of pressure vessel in 1.5 working pressure (**c**), and 2 working pressure (**d**).

A 20 MPa carbon fiber composite pressure vessel, with the same structure size as the hydrogen storage pressure composite vessel that has a 35 MPa metal liner, was used as the experimental object. The vessel had a hemispherical head column and a pipe mouth at the top. The volume of the vessel was approximately 50 L, the inner lining material was 6061-T6 aluminum alloy, and the fiber wound on the outside was T700 carbon fiber. There was a total of eight sensors: six sensors were placed in the cylindrical part of the vessel (each three sensors around the vessel), and two sensors were situated in the upper and lower heads. Figure 3a shows the test vessel with the installed sensors and preamplifiers. The layout of the sensors on the composite pressure vessel is shown in Figure 3b, where #2 and #5 are R15α sensors (their frequency range is 75–150 kHz) monitoring damage to the metal lining, and #1, #3, #4, #6, #7, and #8 are WD sensors, which monitor the damage of composite structure of the vessel.

According to the design and manufacturing parameters of the pressure vessel, the pressure vessel hydraulic test loading method (Chapter 11 T-1142c-2-an of ASME Boiler and pressure vessel Code V) was used and two maximum pressures of 30 (1.5 times working pressure) and 40 MPa (2 times working pressure) were set for loading tests. The loading curve is shown in Figure 3c,d, the pressure increase is 3 MPa/time, and the time of each pressure holding stage is 4 min.

### 2.4. Analytical Method

#### 2.4.1. Empirical Mode Decomposition (EMD) Theory

EMD starts from the local characteristics of the signal and directly constructs the basis function from the signal to obtain decomposition components of different scales. In particular, after the introduction of resolution, EMD is used to overcome the possible modal aliasing phenomenon in signal decomposition. EMD is employed to decompose a complex signal into a number of intrinsic mode functions (IMFs). It is based on a basic assumption [31,32] that any complex signal is composed of some different IMFs, and each IMF, whether linear, nonlinear, or nonstationary, has the same number of extreme points and zero-crossing points. There is only one extreme point between two adjacent zero-crossing points. Moreover, the upper and lower envelopes are locally symmetric about the time axis, and any two modes are independent of each other; a signal can contain multiple IMFs at any time. If the modal functions overlap with each other, a complex signal is formed. Based on this assumption, the EMD method can be used to decompose any signal *s*(*t*).

For any signal *s*(*t*), first, all the local extreme points of the signal should be determined; then, all the local maximum points should be connected by cubic splines to form the upper envelope. Subsequently, all the local minimum points should be connected by cubic splines to form the lower envelope. The upper and lower envelopes should envelop all the data points. The upper envelope is written as *u*_0_(*t*) and lower envelope is written as *v*_0_(*t*) of *s*(*t*).

The mean curve of the upper and lower envelopes is recorded as *m*_0_(*t*), that is:

(1) $$m_{0}(t)=\frac{u_{0}(t)+v_{0}(t)}{2}$$

(2) $$h_{1}(t)=s(t)-\;m_{0}(t)$$

If *h*_1_(*t*) satisfies the two IMF criteria above, then the first IMF component *c*_1_(*t*) is obtained and given by *c*_1_(*t*) *= h*_1_(*t*). Otherwise, *h*_1_(*t*) is treated as a new datum in the subsequent sifting process. The sifting process is repeated, considering *h*_1p_(*t*) as:

(3) $$h_{1p}(t)=h_{1(p-1)}(t)-m_{1p}(t)$$

If *h*_1p_(*t*) satisfies the two IMF criteria above, then the first IMF component *c*_1_(*t*) is *c*_1_(*t*) *= h*_1p_(*t*).

The residue *r*_1_(*t*) is defined as:

(4) $$r_{1}(t)=s(t)\;-\;c_{1}(t)$$

This parameter is treated as the new data to be subjected to the same sifting process described above and will be used to obtain the second IMF component *c*_2_(*t*).

The sifting process is finished when one of these conditions is met: (1) the residue *r*_n_(*t*) becomes a monotonic function; (2) *c*_n_(*t*) or *r*_n_(*t*) is less than a predetermined value. Thus, the original signal *s*(*t*) can be expressed in terms of IMF components *c*_i_(*t*) (i = 1, 2, …, n) and final residue *r*_n_(*t*) as follows:

(5) $$\;s(t)=\overset{m}{\underset{i=1}{\sum }}c_{i}(t)+r_{n}(t)$$

The decomposition process of EMD is actually a “modal separation” process. In the “modal separation” process, the superposition of modal waveforms is eliminated, and the waveform profile is highly symmetrical.

#### 2.4.2. Damage Modal Characteristics of Composite Material

**Figure 4:**
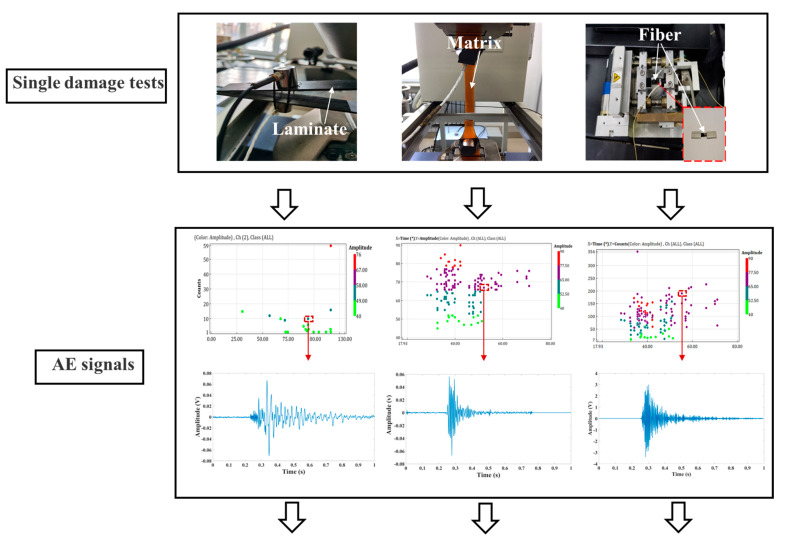

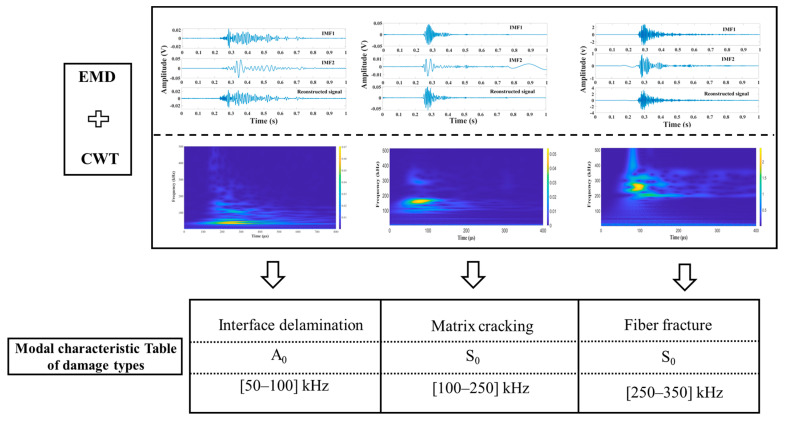
Damage modal feature extraction.

To extract the modal characteristics of the three main damage forms of composite materials, three damage tests (delamination test, matrix cracking test, fiber breaking test) were used. Due to the word limitation of the paper, the details of three experimental procedures are not presented here. The materials used in the test were carbon fiber laminates, epoxy resin, and fiber bundles. Epoxy resin and fiber bundles were the same as the matrix and fiber materials of the carbon fiber composite pressure vessel. Through the EMD decomposition of AE signals collected during damage, all the IMFs of the three types of damage signals were obtained. Because EMD decomposition is an adaptive decomposition method using time as the characteristic scale, the modes of a certain scale range are reflected by each IMF, and modal aliases do not appear. In the frequency domain, IMFs with different scales show orderly arrangement from high to low frequencies. IMF1-2 retained the details of the original signal well, and the frequency component was high and accounted for a large proportion in all the frequencies of the original signal. To extract the characteristic parameters of AE signal accurately and exclude the interference from the mode that aliased low-frequency components in the signal, IMF1 and IMF2 were selected and reconstructed through EMD decomposition. Finally, the continuous wavelet transform (CWT) was performed on the reconstructed signals, and the main modal components and frequency distribution range of AE signals with three damage forms were observed. A complex Morlet wavelet was selected for CWT because it could be used to separate the amplitude and phase and had high accuracy in frequency analyses [33,34]. Figure 4 shows the extraction flow chart of the modal characteristics of damage of the three composite materials.

#### 2.4.3. MAE Parameter Establishment

**Figure 5:**
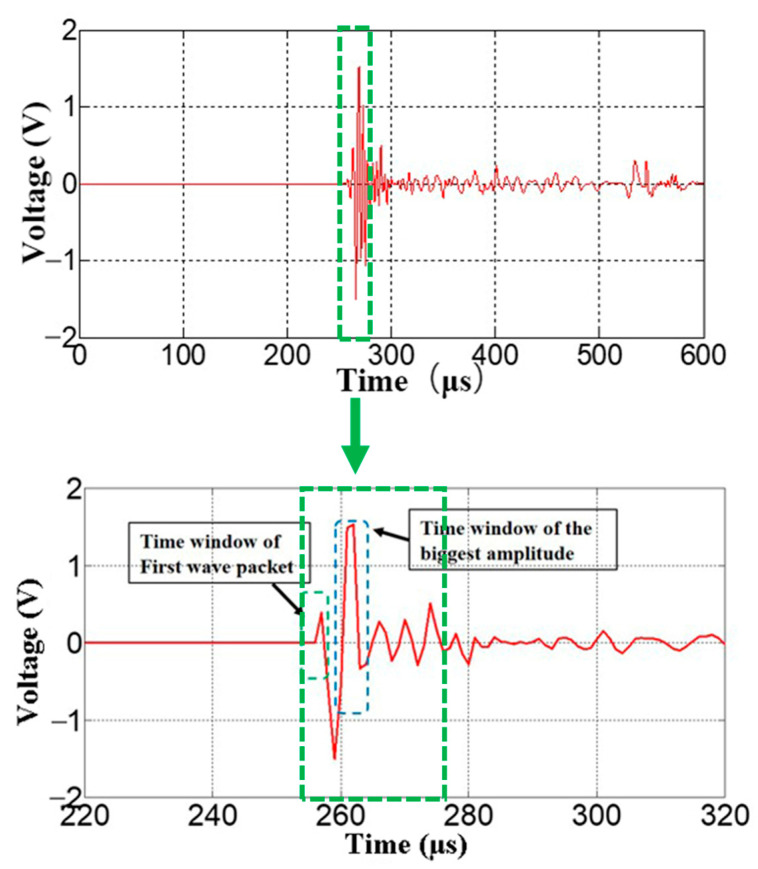
Local detail extraction of the signal inside the green box line.

It can be seen from the time-domain waveforms of the modal components that the arrival time of the *S*_0_ mode is shorter than that of the A_0_ mode, and the two waveform modes overlap in the time domain; thus, to improve the recognition resolution of the two modes of all components, an appropriate window must be selected to extract the local details of the signal [35]. Then, the differentiated waveform of the modal component can be obtained. If the waveform amplitude in the time window is considered a parameter, the transient amplitude of this mode can be identified quantitatively. For the selection of the time domain window, according to the specific characteristics of the waveform, the modal waveform packet in the window is used as the main reference. The width of the window is determined according to the effect of modal separation. The basic principle is based on the first wave packet of the modal waveform. If the first wave packet is difficult to extract, it is extended to the wave packet with the largest amplitude. The window selection process is shown in Figure 5. The specific steps used for selecting a specific window are as follows:

(1) Set the time domain window according to the arrival time of the modal transient waveform distribution;
(2) Perform modal separation on the signal to obtain the modal component waveforms distributed under the time domain window;
(3) The first wave packet is the priority selection object. If the amplitude of the first wave packet is less than 1/10 of the peak amplitude, select the wave packet with the largest amplitude.

After the time window is selected, the MAE mode waveform can be quantified numerically by extracting the characteristic parameters of a simplified AE waveform. According to the characteristics of the AE signal of composite material damage, the maximum amplitude of the wave packet in the window is determined as the window time amplitude (WTA). The mathematical model of WTA is shown in Formula (6):

(6) $$\mathrm{WTA}=\max_{t=t_{1}}^{t_{2}}\{\left|\mathrm{MAE}\right|\left[V_{n}\left(t\right)\right]\}$$

With reference to the absolute energy of the traditional AE signal, the absolute energy of the wave packet in the window is defined as the window time energy (WTE). The mathematical model of WTE is shown in Formula (7):

(7) $$\mathrm{WTE}=E\int V^{2}dt/Z$$

where *V* is the signal amplitude; Z is the input impedance of the preamplifier, and the input impedance refers to the equivalent impedance of the input end of a circuit; and *E* is the correction coefficient between the damage mechanical energy of the composite material and AE waveform energy.

According to the MAE parameters established and the damage modal characteristics presented in Section 2.4.2, the damage discrimination Formula (8) is obtained as follows:

(8) $${R=\mathrm{WTA}}_{A_{0}}-{\mathrm{WTA}}_{S_{0}}$$

When ${\;\mathrm{WTA}}_{A_{0}}$ > ${\mathrm{WTA}}_{S_{0}}$, the damage is determined as the interface delamination damage. If ${\;\mathrm{WTA}}_{A_{0}}$ < ${\mathrm{WTA}}_{S_{0}}$, two HF bandpass filters are selected as the screening criteria. The frequency center of S_0_ modal component located at [100–250] kHz is determined as matrix cracking damage, and that of S_0_ modal component located at [250–350] kHz is determined as fiber breakage damage.

## 3. Results and Discussion

### 3.1. Verification of the Modal Separation Method

**Figure 6:**
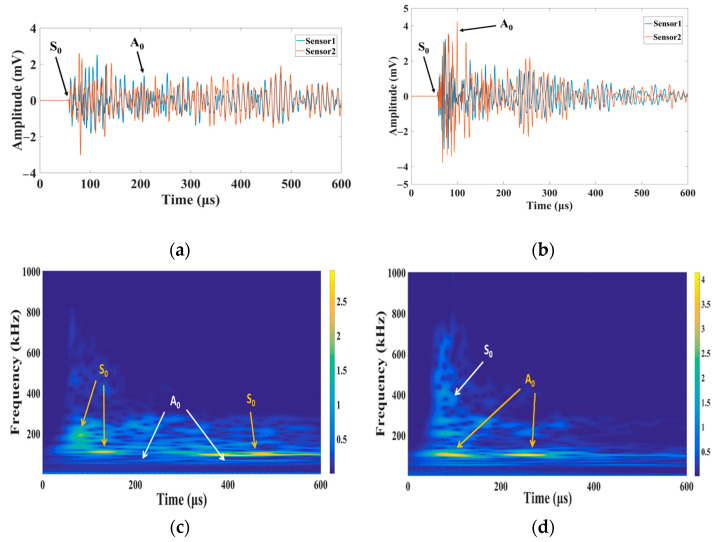
AE signal diagram: extracted lamb mode IP source (**a**) and OP source (**b**); CWT diagram: IP source (**c**) and OP source (**d**).

Figure 6a,b display the original signal without any filtering for the two methods of PLBs; although the two modes can be visually discerned (signals of the S_1_ and S_2_ sensors are in the same phase in the S_0_ mode, whereas in the A_0_ mode, those of these sensors are in opposite phases), data processing is required if they are separated. Therefore, the continuous wavelet transform is used to perform the continuous wavelet transform off-line. The obtained side-broken lead signal spectrum is shown in Figure 6c. The frequency is mainly distributed between 100–300 kHz. In the spectral diagram of continuous wavelet transform, the low-order spread wave S_0_ with a frequency of approximately 100 kHz coincides with A_0_, but the time range of its frequency distribution is larger than that of A_0_; that is, the S_0_ mode occupies the main part in the time domain, and its energy is higher than that of the A_0_ mode. The S_0_ mode is dominant in the IP source. The continuous wavelet transform spectrum of surface lead breaking is shown in Figure 6d, and the frequency range is mainly distributed between 100–300 kHz. The frequency in Figure 6d coincides with S_0_ in the spread wave A_0_ near 100 kHz, but the time duration of frequency distribution between100–200 kHz is longer. A_0_ occupies the main part in the time domain, and its energy is higher than that of the S_0_ mode; thus, the A_0_ mode is dominant in the OP source. Therefore, it can be seen from the lead breaking experiment that the S_0_ and A_0_ modes are dominant in the IP and OP sources, respectively.

**Figure 7:**
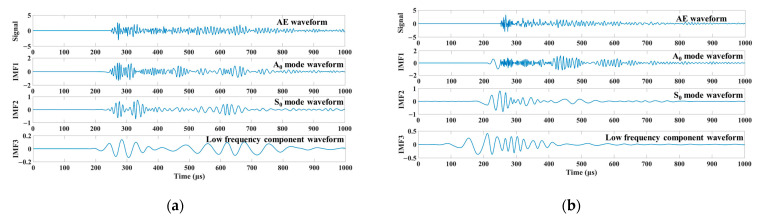
Signal modal component waveform diagram: IP source (**a**), OP source (**b**).

The AE signals of the two sound sources in the lead breaking experiment were processed using the EMD method in MATLAB. The process is similar to the analysis program presented in Section 3.2. All the IMFs were obtained through the EMD decomposition of signals. IMF1-2 retained the details of the original signal, exhibited a high frequency component, and accounted for a large proportion in all the frequencies of the original signal, and its frequency distribution corresponded to S_0_ and A_0_. Therefore, IMF1-2 was selected as the corresponding modal waveform (Figure 7). Although both S_0_ and A_0_ modes are included in the modal components of the IP and OP sources, the amplitudes of the modal components are different. In the IP source, the S_0_ modal amplitude of the signal is high, which occupies the main part of the signal component. The A_0_ mode is the main component of the OP source, and the amplitude of the S_0_ mode is considerably lower than that of the A_0_ mode. This result reveals that the in-plane excitation source propagates mainly in the form of spreading waves, and the out-of-plane excitation is mainly propagated in the form of bending waves, which is consistent with the propagation mechanism of Lamb waves and verifies the accuracy of the proposed modal separation method.

### 3.2. Mode Analysis of Delamination Damage

**Figure 8:**
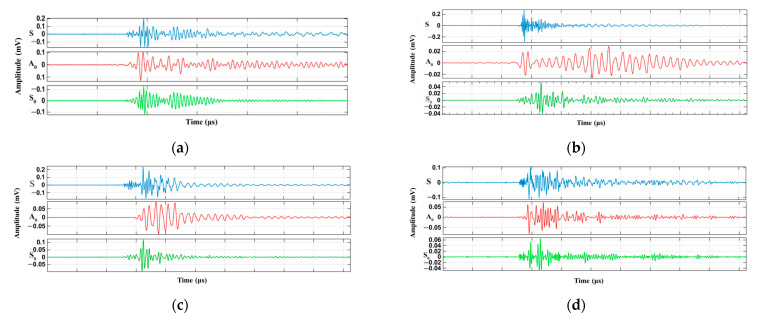

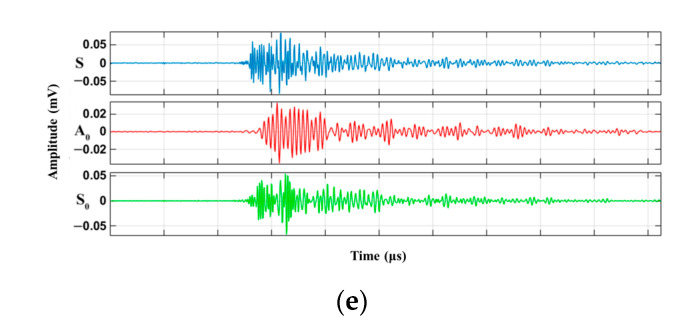
Waveform diagrams of specimens in different load modes: P_1_–P_5_ stages (**a**–**e**).

By using the EMD mode separation method presented in Section 3.1, the modal characteristics of the AE signals generated during the loading of laminates were separated, and the modal characteristics of signals S_0_ and A_0_ at each stage of P_1_–P_5_ were extracted. The results of different stages of loading according to Figure 2b are shown in Figure 8a–e. It can be seen from Figure 8a–e that the modal characteristics of all the five stages P_1_–P_5_ include S_0_ and A_0_ modes, and there are obvious differences between the two types of modes, but the complete waveform always has a certain degree of reflection and mode conversion effects. Therefore, compared with the peak amplitude, the amplitude of the signal A_0_ mode in the P_1_–P_5_ stage is slightly larger than that of the S_0_ mode. The comparison of the peak amplitude shows that the bending wave component is dominant in the mode.

**Figure 9:**
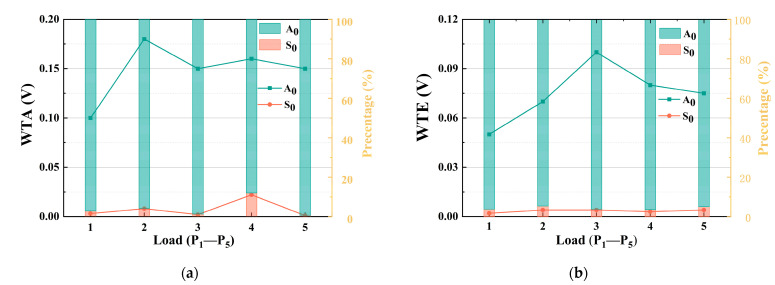
Comparison of MAE characteristic parameters between WTA (**a**) and WTE (**b**).

To effectively improve the resolution of modal separation, the waveform reflection is eliminated by selecting a time window with reference to the modal AE parameters, and the WTA and WTE parameters corresponding to the load level are extracted (Figure 9). Figure 9 shows the numerical trend of the WTA and WTE characteristic parameters of the A_0_ and S_0_ modes under different load levels. The values of the WTA and WTE characteristic parameters of the A_0_ mode are considerably higher than those of the S_0_ mode, and the value difference is approximately 10 times. During the bending of the carbon fiber composite material, the delamination damage signal mainly propagates in the A_0_ mode. Combined with the results of the aforementioned analysis using the EMD method, it can be determined that the mode of the delamination damage signal is dominated by bending waves.

### 3.3. Damage Identification and Evaluation of Carbon Fiber Composite Pressure Vessels

For the carbon fiber composite pressure vessel, first, the conventional AE parameter analysis was performed, and the damage evolution law was preliminarily analyzed; however, accurate damage type identification could not be achieved. Therefore, the method based on modal AE was used to analyze the vessel, and the three types of damage were characterized according to the established damage discrimination method, which is consistent with the test method. Finally, the established parameters, WTE and WTA, were used to process the damage signal of fiber fracture, and the change in damage amount caused by pressure change was determined sensitively.

**Figure 10:**
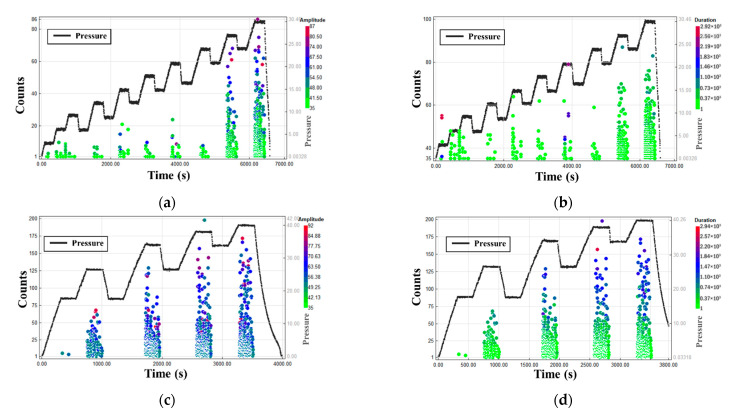
Time–counts–amplitude diagram: 0–30 (**a**) and 0–40 MPa (**c**); Time–counts–duration diagram: 0–30 (**b**) and 0–40 MPa (**d**).

For the hydraulic loading test of the pressure vessel in the range of 0–30 and 0–40 MPa, the AE parameter analysis diagram is shown in Figure 10. From the distribution of the counts in the time domain, when the vessel pressure is <20 MPa, the AE signal is limited, and the signal change is highly random. When the pressure is >20 MPa, the AE signal considerably enhances and becomes correlated with the amplitude and duration. A few high-amplitude (>70 dB) signals appear, but the duration is <1000 μs. A combination of the characteristics of the damage parameters shows that the signal characteristics are highly consistent with matrix cracking and interface delamination. However, the correlation between amplitude and duration leads to the overlapping of signals; thus, classifying the types of damage is considerably difficult.

Compared with Figure 10a,c, the counting rate of loading tests of the pressure vessel in the range of 0–40 MPa increases considerably during pressurization, and it continues to increase after pressurization exceeds 30 MPa. The high amplitude signal increases obviously, and the increment rate of signal quantity increases because the increase in pressure further expands the original accumulated damage during continued pressurization. Through the data analysis of the pressure vessel water pressure experiment at the working pressure of >20 MPa, the results revealed that pressure vessel damage gradually appeared with the pressurization process, and a corresponding relationship existed between the damage state and the type of damage inside the vessel and change law of the AE signal. Therefore, the changing status of different damage types can be obtained through the parameter value range and proportion distribution of the AE signal, but the classification boundary is not clear. Distinguishing damage types only from the numerical of the parameter range can lead to inaccurate selection. To improve the classification accuracy, the MAE method was used to analyze the damage pattern and the signal evolution law of each damage type.

**Figure 11:**
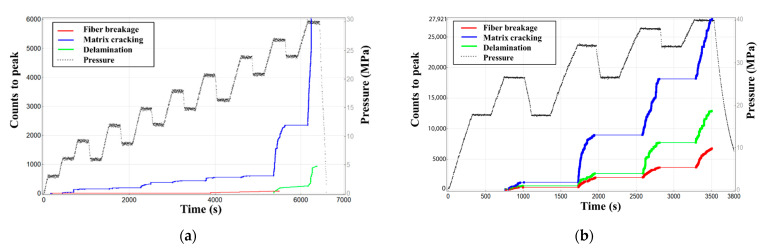
Damage mode evolution diagram of AE signal in hydraulic test of 0–30 (**a**) and 0–40 MPa (**b**).

Based on the damage criterion algorithm presented in Section 3.3, the AE signal of the vessel is loaded into the 0–30 and 0–40 MPa hydraulic pressure tests for modal separation, and the separation result is shown in Figure 11. The algorithm can effectively realize the accurate identification of three damage types (fiber fracture, matrix cracking, and interface delamination). The matrix cracking signal increases considerably at the pressure of >20 MPa, and the interface delamination damage and fiber fracture damage signals increase slightly. The total amount of the matrix cracking signal during the loading process exceeds other signals, but the fiber breaking signal mainly appears under high load. The amount of fiber breaking signal before the final vessel failure is considerably smaller than other damage modes. Figure 11b shows that the fiber fracture signal increases in the high load part above 30 MPa. Compared with the parametric analysis, the aforementioned analysis results can realize the effective separation of different damage types and are able to characterize the law of damage evolution.

**Figure 12:**
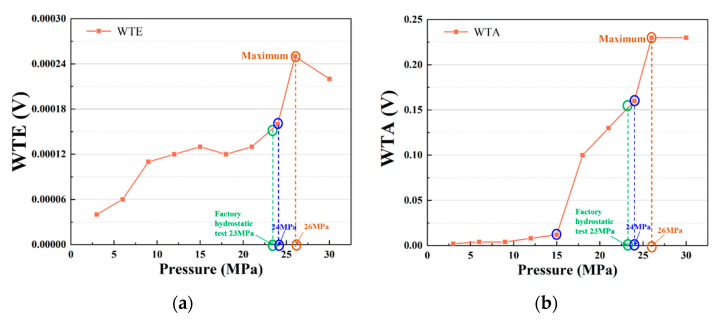
Correlation of fiber fracture characteristic parameters: WTE (**a**) and WTA load (**b**).

According to the damage mechanism of composite material vessels, numerous fiber fractures indicate an overall serious failure trend of the structure [36,37]. The experimental results revealed that the increase in fiber fracture damage did not lead to the overall instability of the vessel structure, which indicated a certain cumulative threshold of fiber fracture damage. Therefore, the evaluation of the damage state of pressure vessels can be based on the fiber fracture damage threshold, and the fiber fracture damage threshold must be used to establish the corresponding relationship between the threshold parameters and load. The corresponding relationship between the fiber fracture characteristic parameters WTA and WTE of MAE and the load is established as shown in Figure 12.

Figure 12a reveals that the point (inside the blue circle) of the increase in WTE appears after the working pressure of 20 MPa; that is, WTE increases slowly before 24 MPa. WTE increases considerably after 24 MPa and reaches the maximum value at 26 MPa. The pressure vessel manufacturing process is subjected to a hydraulic test before leaving the factory. The general test pressure is 1.15 times of the working pressure for 23 MPa. Because the pressure of the pressure vessel reaches 23 MPa before the monitoring experiment, the vessel may already exhibit stress concentration or minor damage before the experiment, which is consistent with the slight increase in the WTE value before the working pressure exceeds 23 MPa. However, after pressure exceeds 24 MPa, the WTE value increases obviously. This result shows that WTE is highly sensitive for damage identification. The distribution of WTA parameters with a load is presented in Figure 12b, and its increasing trend exhibits obvious abrupt characteristics. It can be seen that a change in the increasing trend appears at approximately 15 MPa, and two points exist. The second point appears at 24 MPa. The second point is consistent with the position of the WTE parameter, which reveals that WTA exhibits an excellent characterization effect on damage changes.

## 4. Conclusions

The study mainly used the MAE method to analyze the damage mode of the carbon fiber composite pressure vessel during the hydraulic test. The modal separation method, based on EMD, was verified using preliminary PLBs and bending tests. The damage modal characteristics of composite materials were obtained by employing single damage tests, and the waveforms were determined by selecting the appropriate window. WTA and WTE parameters were established in this window. The damage modal characteristics of composite materials and WTA and WTE parameters were selected to determine the identification method of the damage mode. A hydraulic test was performed on the vessel, and AE signals were detected by using the multi-step loading method. The delamination, matrix cracking, and fiber fracture modes were recognized in the damage signal. Moreover, the correlation between WTA and WTE for the fiber fracture damage threshold was studied during hydraulic test. The main conclusions obtained are as follows:

(1) The proposed modal separation method is accurate. The damage signal can be analyzed using this method in the bending AE monitoring experiment of carbon fiber composite laminate. The AE signal source of delamination damage produces excitation similar to that of in-plane action, mainly A_0_ mode propagation.
(2) A comparison of the MAE parameters WTA and WTE of the signal in the bending test and load correlation showed that the A_0_ mode is dominant in the delamination damage; this finding is the same as the conclusion of the modal separation method. This shows that the establishment of WTA and WTE parameters is reliable.
(3) The modal feature criterion algorithm can more accurately identify the three types of damage caused by carbon fiber composite vessels during pressurization than the analysis of AE parameters. Because there is a certain cumulative threshold of fiber fracture damage, the evaluation of the damage state of pressure vessels can be based on the threshold of fiber fracture damage, and the damage process can be well characterized using WTE and WTA.

## Figures and Tables

**Table 1:** Parameter setting of the AE monitoring system.

Project	Channel	Threshold (dB)	Sampling Rate (MSPS)	Pre-Trigger (μs)	PDT (μs)	HDT (μs)	HLT (μs)
parameter	2	35	2	256	100	200	400

## Data Availability

Data can be provided upon request from the correspondence author.
